# The Role of Concern over Mistakes in the Association Between Social Anxiety and Disordered Eating Behaviours: A Structural Equation Modelling Study

**DOI:** 10.3390/ejihpe16100163

**Published:** 2026-10-14

**Authors:** María J. Triguero-López, M. Inmaculada Moreno-García, Manuel Morales-Ortiz

**Affiliations:** 1 Department of Personality, Assessment and Psychological Treatments, Faculty of Psychology, University of Seville, Calle Camilo José Cela, 41018 Seville, Spain; imgarcia@us.es; 2 Department of Experimental Psychology, Faculty of Psychology, University of Seville, Calle Camilo José Cela, 41018 Seville, Spain; morales@us.es

**Keywords:** social anxiety, disordered eating, concern over mistakes, perfectionism, structural equation modelling

## Abstract

Social anxiety and disordered eating frequently co-occur, but the psychological processes underlying this association remain unclear. This study examined concern over mistakes, a core dimension of maladaptive perfectionism, as a candidate shared transdiagnostic correlate of both domains. A cross-sectional sample of 890 participants aged 18–25 years (M = 20.02, SD = 1.94), including 338 women (38.0%), 546 men (61.3%), and 6 participants (0.7%) who did not report their sex, from vocational training and university settings in Spain, completed measures of social anxiety, disordered eating behaviours, and concern over mistakes. Structural equation modelling was used to examine two alternative structural specifications differing in the direction of the association between social anxiety symptoms and disordered eating behaviours. Both models showed acceptable and very similar descriptive fit (Model A: CFI = 0.947, TLI = 0.947, RMSEA = 0.044, SRMR = 0.064; Model B: CFI = 0.946, TLI = 0.947, RMSEA = 0.044, SRMR = 0.064). Across both alternative structural specifications, the associations among social anxiety symptoms, disordered eating behaviours, and concern over mistakes were statistically significant, including the indirect associations involving concern over mistakes. These findings suggest that concern over mistakes may represent a shared transdiagnostic cognitive correlate associated with both social anxiety symptoms and disordered eating, without supporting a specific causal direction.

## 1. Introduction

Social anxiety and eating disorders show a high rate of co-occurrence in both clinical and community samples (Kerr-Gaffney et al., 2018). This pattern has been associated with greater clinical severity, poorer prognosis, and increased therapeutic difficulties (Vall & Wade, 2016), which has led to growing interest in identifying the psychological processes underlying the relationship between these two domains (Hofmann, 2024). This co-occurrence has been documented in both cross-sectional and longitudinal studies and has been linked to barriers to treatment access, greater symptom persistence, and poorer clinical outcomes (Goodwin & Fitzgibbon, 2002; Levinson et al., 2018; Trompeter et al., 2025; Vall & Wade, 2016).

Several studies have suggested that the association between social anxiety and eating disorders may be explained, at least in part, by shared psychological vulnerabilities (Morgan-Lowes et al., 2019). In particular, evaluative fears, including social appearance anxiety and fear of negative evaluation, have been consistently associated with both social anxiety symptoms and eating disorder symptomatology (Liu et al., 2022; Wong et al., 2021), supporting the presence of common processes of social self-evaluation (Levinson et al., 2013; Menatti et al., 2015; Merve et al., 2025). However, these evaluative fears are often embedded within broader cognitive frameworks, among which maladaptive perfectionism has been conceptualised as a relevant transdiagnostic process (Egan et al., 2011; Limburg et al., 2017). Prospective and advanced modelling studies suggest that these evaluative fears form part of a network of shared vulnerabilities that jointly contribute to social anxiety and eating disorder symptomatology (Brosof et al., 2019; Levinson & Rodebaugh, 2016; Bottera et al., 2024).

Within this framework, not all dimensions of perfectionism exhibit the same level of psychopathological relevance. From a multidimensional perspective, concern over mistakes occupies a particularly prominent position due to its association with self-criticism, sensitivity to failure, and the anticipation of negative consequences following imperfect performance (Frost et al., 1990; Ralph-Nearman et al., 2024; Smith et al., 2022). Accumulated evidence indicates that perfectionistic concerns are robustly associated with both social anxiety and eating disorder symptomatology in clinical and non-clinical samples (Cox & Chen, 2015; Ferber et al., 2024; Jain & Sudhir, 2010; Kehayes et al., 2019; Stackpole et al., 2023; Wang et al., 2022). From a transdiagnostic perspective, concern over mistakes, as suggested by Smith et al. (2022), can be understood as a cognitive process of contingent self-evaluation that integrates error sensitivity, negative social evaluation, and control behaviours implicated in both domains.

Despite this evidence, relatively few studies have examined in an integrated manner the specific role of concern over mistakes in the relationship between social anxiety symptoms and disordered eating behaviours. Those that have done so have primarily conceptualised it as a causal mediator between these difficulties (Levinson et al., 2018), and even fewer have tested alternative structural specifications that allow the exploration of plausible directions of association (Drieberg et al., 2019). The comparison of alternative models using structural equation modelling (SEM) represents a valuable methodological strategy, as it allows different patterns of direct and indirect association under alternative directional specifications to be examined without assuming a priori a single causal direction. Methodological studies have highlighted the importance of this approach when conducting mediation analyses with cross-sectional observational data (Ansah et al., 2024; Huh et al., 2022).

Moreover, reviews on mediation methods in observational research emphasise that traditional approaches (typically centred on strict causal mediation) are frequently employed but rarely consider alternative structural specifications or model comparisons that could evaluate the robustness of the observed relationships (Rijnhart et al., 2021).

Recent studies indicate that concern over mistakes and other perfectionistic concerns function as central and bridging symptoms, linking perfectionism with anxiety symptoms, self-esteem, and eating-related difficulties in both adolescents and adults (Delaquis et al., 2024; Livet et al., 2023). This evidence aligns with theoretical perspectives suggesting that psychological symptoms are organised in interconnected networks in which certain nodes may perform central and transdiagnostic functions (Borsboom, 2017).

In this context, it is particularly relevant to adopt analytical approaches that avoid unidirectional causal inferences while allowing the estimation of patterns of direct and indirect association consistent with shared psychological processes (Levinson et al., 2024; Levinson & Rodebaugh, 2012). In line with recent prospective reviews suggesting associations compatible with alternative directions between anxiety and eating-related symptoms over time (Trompeter et al., 2025), the comparison of alternative structural models allows results to be interpreted with greater methodological caution and theoretical coherence. Accordingly, an important gap remains regarding whether concern over mistakes is consistently associated with both social anxiety symptoms and disordered eating behaviours across alternative structural specifications that do not assume a single causal direction.

The present study aimed to examine the structural associations between symptoms of social anxiety, disordered eating behaviours, and concern over mistakes using structural equation modelling. Specifically, the objectives were:

1. To examine the direct association between symptoms of social anxiety and disordered eating behaviours.
2. To examine whether concern over mistakes is consistently involved in the statistical association between both constructs across alternative model specifications.
3. To compare alternative structural specifications in order to assess the robustness of the observed patterns of association.

Based on previous evidence showing associations between social anxiety and eating-related symptoms (Levinson & Rodebaugh, 2012, 2016; Trompeter et al., 2025), between perfectionistic concerns and both anxiety and eating-related symptomatology (Smith et al., 2022; Stackpole et al., 2023), and suggesting a role for shared vulnerabilities in the association between social anxiety and eating-disorder symptoms (Levinson et al., 2018), the following hypotheses were formulated:

**H1.**  *Social anxiety symptoms and disordered eating behaviours will be positively associated.*

**H2.**  *Concern over mistakes will be positively associated with both social anxiety symptoms and disordered eating behaviours.*

**H3.**  *Significant indirect associations involving concern over mistakes will be observed across both alternative structural specifications.*

## 2. Materials and Methods

### 2.1. Participants

The study employed a cross-sectional quantitative design and was conducted in the province of Seville, Spain. The sample consisted of 890 students aged between 18 and 25 years who, at the time of the study, were enrolled in vocational training programmes (intermediate and advanced levels) and university studies (*M* = 20.02; *SD* = 1.94). Of these, 338 identified as female (38%, *M* = 20.21; *SD* = 1.89), 546 as male (61.3%, *M* = 19.91; *SD* = 1.97), and 6 participants (0.7%) did not report their sex. Participants were recruited from three educational institutions in Seville, Spain: two vocational training centres and the University of Seville. The initial dataset comprised 890 participants. Following data screening, 185 questionnaires were excluded because of missing responses, resulting in a final analytical sample of 705 participants for the CFA and SEM analyses. The exact number of eligible students approached, and the corresponding refusal rate, were not systematically recorded and therefore could not be determined reliably.

The minimum required sample size was calculated prior to data collection using the standard formula for large or unknown populations, assuming a 95% confidence level (*z* = 1.96), maximum variability (*p* = 0.50), and a 5% margin of error (*e* = 0.05). The resulting minimum sample size was 384.16 participants, rounded up to 385. The analytical sample comprised 705 complete cases. Sample adequacy was considered in relation to the complexity of the models and the ordinal estimation approach rather than on the basis of a general rule of thumb. The reference measurement model included 40 ordinal indicators across three latent variables, 65 freely estimated model parameters excluding item thresholds, and 715 degrees of freedom; each structural model contained 67 freely estimated model parameters excluding item thresholds and 793 degrees of freedom. Item thresholds were additional freely estimated parameters under the ordinal specification and are not included in these counts. Methodological research indicates that sample-size requirements in SEM depend on characteristics such as model complexity, number of indicators, factor loadings, and estimation method (Rhemtulla et al., 2012; Wolf et al., 2013). Participants were selected through non-probabilistic convenience sampling. Educational institutions were chosen according to two criteria: (a) having students within the required age range and (b) granting institutional permission for participation. Only students from institutions meeting both criteria were included in the study.

The inclusion criteria were: (1) being enrolled in a secondary-level vocational, advanced vocational, or university programme; (2) being between 18 and 25 years of age; and (3) completing the entire assessment protocol. The exclusion criteria were: (1) not being enrolled in an educational institution at the time of the study; (2) being younger than 18 or older than 25 years; and (3) submitting incomplete questionnaires. The study was approved by the Ethics Committee of the University of Seville (protocol code 2024-1448) and adhered to the principles of the Declaration of Helsinki (World Medical Association, 2013).

### 2.2. Procedure

Data collection was conducted collectively in classrooms at the participating institutions. Prior to the beginning of the assessment session, each participant received an information sheet, an informed consent form, and the study questionnaires. The objectives of the research and the instructions for completing the instruments were explained in detail. All participants provided written informed consent. Data collection was conducted from June to October 2024.

The assessment session lasted approximately 40 min. Administration was supervised by the researchers, who answered participants’ questions and ensured that the inclusion criteria were met.

### 2.3. Instruments

The Eating Attitudes Test (EAT-26) (Garner & Garfinkel, 1979) (*α* = 0.90) is a 26-item self-report instrument that assesses attitudes and behaviours related to eating and eating-disorder symptomatology. Higher scores indicate greater levels of disordered eating attitudes and behaviours. Previous psychometric research has shown that the original factorial structure of the EAT-26 may vary across populations and that alternative item configurations can provide better fit than the original specification (Bazo Perez et al., 2025). During the preliminary adaptation stage, 12 items from the original EAT-26 were retained (original items 1, 2, 4, 6, 7, 10, 11, 14, 16, 17, 18, and 21). The remaining 14 original items (3, 5, 8, 9, 12, 13, 15, 19, 20, 22, 23, 24, 25, and 26) were not included in the CFA. In the analytical dataset, the retained items were relabelled TCA1–TCA12 (Table S1). Administered in Spanish, the EAT-26 has demonstrated reliability and validity in Spanish samples (Rivas et al., 2010). However, the present study used a study-specific reduced item set derived from the EAT-26 rather than the complete validated instrument.

Frost’s Multidimensional Perfectionism Scale (FMPS) (Frost et al., 1990) (*α* = 0.87–0.93). The FMPS was administered in Spanish and has been validated for use in the Spanish population (Carrasco et al., 2009). For the purposes of the present study, concern over mistakes (COM), a specific dimension of the FMPS, was examined. This dimension reflects negative reactions to mistakes, the tendency to interpret mistakes as failures, and heightened concern about the consequences of imperfect performance. Previous research has shown that abbreviated FMPS forms can be developed by retaining the strongest-loading indicators within each dimension and subsequently confirming the reduced structure through CFA (Soares et al., 2017). For the present study, four indicators were retained to represent the Concern over Mistakes dimension (P9_PRE, P12_PRE, P13_PRE, and P14_PRE) (Table S1). The preliminary adaptation retained four items from the original Concern over Mistakes dimension (original FMPS items 9, 13, 14, and 18), which corresponded to P9_PRE, P12_PRE, P13_PRE, and P14_PRE in the reduced administered questionnaire. Other FMPS items were not specified as indicators of the COM latent construct. Accordingly, the present study used a reduced item set from the Concern over Mistakes dimension rather than the complete validated Spanish version.

Liebowitz’s Social Anxiety Scale (LSAS) (Liebowitz, 1987) comprises 24 situations that are assessed in terms of both fear/anxiety and avoidance and that are typically problematic for individuals with social anxiety. Each item assesses both fear and avoidance. Previous psychometric studies have shown that fear and avoidance ratings may exhibit more complex factorial structures than the original specification (Safren et al., 1999; Zubeidat et al., 2008). For the present analyses, two LSAS subscales were used: the Fear subscale (AS_MI), which assesses the degree of fear in social situations, and the Avoidance subscale (AS_EV), which measures the tendency to avoid such situations. In the present measurement model, the retained fear and avoidance indicators were specified as reflective indicators of a single latent SA factor, because both response domains were used to represent the broader construct of social anxiety symptoms rather than modelled as separate outcomes. A reduced set of 25 LSAS responses was entered into the initial CFA, comprising 13 fear ratings (situations 1, 3, 4, 6, 7, 9, 10, 12, 15, 17, 18, 19, and 20) and 12 avoidance ratings (situations 2, 3, 4, 5, 7, 8, 9, 11, 12, 13, 14, and 16). The remaining fear and avoidance ratings from the original 24-situation LSAS were not entered into the CFA. During CFA evaluation, AS_EV14 was subsequently removed because its association with the latent SA factor was 0.246, below the 0.30 criterion applied for retention (Table S1). The questionnaire was administered in Spanish. Previous research has supported the psychometric properties of the LSAS in Spanish-speaking populations, including participants from Spain (Caballo et al., 2019). However, the administered form in the present study was a study-specific reduced adaptation rather than the complete validated LSAS.

### 2.4. Statistical Analysis

To examine the relationships between disordered eating behaviours (ED), social anxiety symptoms (SA), and concern over mistakes (COM), structural equation modelling (SEM) was conducted using the R statistical programme. Firstly, an initial measurement model including three latent variables (ED, SA, and COM) was specified. The initial CFA contained 41 reflective indicators: 12 indicators for ED (TCA1–TCA12), four indicators for COM (P9_PRE, P12_PRE, P13_PRE, and P14_PRE), and 25 indicators for SA. The latter comprised 13 fear indicators (AS_MI1, AS_MI3, AS_MI4, AS_MI6, AS_MI7, AS_MI9, AS_MI10, AS_MI12, AS_MI15, AS_MI17, AS_MI18, AS_MI19, and AS_MI20) and 12 avoidance indicators (AS_EV2, AS_EV3, AS_EV4, AS_EV5, AS_EV7, AS_EV8, AS_EV9, AS_EV11, AS_EV12, AS_EV13, AS_EV14, and AS_EV16). Given the ordinal nature of the indicators, reliability in the present sample was summarised using omega (ω) estimated from the CFA model with the compRelSEM function from the semTools package (Jorgensen et al., 2022), specifying ord.scale = TRUE and obs.var = FALSE. Previously published reliability coefficients reported in the description of the instruments refer to earlier validation studies and should not be interpreted as reliability estimates from the present sample. Discriminant validity among latent variables was assessed using the discriminant validity function from the same package.

Two alternative structural models were then estimated using SEM. Models A and B were specified using the same reference measurement model and differed in the direction of the principal structural association. Sex was originally coded as 1 = women and 2 = men. For the structural models, a binary indicator was created with women as the reference category (0 = women, 1 = men). Age and sex were included as covariates of the final outcome in each structural specification. Their inclusion is consistent with previous structural and path-modelling studies in which age and/or sex were incorporated as demographic covariates or predictors in analyses involving anxiety-related or eating-related outcomes (Gilmartin et al., 2023; Zhu et al., 2025). In addition, previous research indicates that social anxiety symptoms and disordered eating behaviours may vary according to age and sex, supporting their substantive relevance as demographic controls in the present analyses (Asher & Aderka, 2018; Jefferies & Ungar, 2020). Thus, age and sex predicted SA in Model A and ED in Model B, corresponding to the final outcome in each specification. This retained the outcome-specific control structure of the submitted models and was not a prespecified adjustment of all structural associations. Both models included the same two covariate paths and therefore had identical model complexity. In both models, COM was specified as a shared latent variable to examine its statistical contribution to the association between SA and ED across alternative structural specifications. The analyses were conducted at the individual level. Because verified classroom- or institution-level identifiers were not retained in the analytical dataset, clustering by classroom or educational institution could not be incorporated into the models. Accordingly, the reported standard errors and confidence intervals rely on the assumption of independence among observations.

Given the cross-sectional nature of the design, the models were interpreted as alternative models without assuming temporal precedence among the constructs. The analyses were conducted using R version 4.3.3. Confirmatory factor analysis (CFA) and structural equation modelling were performed using the lavaan package (version 0.6-17; Rosseel, 2012), and the corresponding reliability and discriminant-validity analyses were conducted using semTools (version 0.5-6). Prior to CFA estimation, preliminary item screening had been conducted using principal-components analysis with Varimax rotation and internal-consistency analysis. Factor loadings and cross-loading patterns were examined to inform item selection, with loadings above 0.50 without substantial cross-loadings providing particularly clear evidence of item-component correspondence. Internal consistency was examined using Cronbach’s alpha. During CFA evaluation, item–factor associations were examined. AS_EV14 was the only indicator removed at this stage because its association with the latent SA factor was 0.246, below the 0.30 criterion applied for retention; no other indicator was removed during CFA evaluation. These item-selection decisions were informed by the observed psychometric results rather than by a preregistered or fully prespecified item-retention rule. Modification indices were examined to identify local areas of model misfit. They were not used to determine item removal. Twenty-two within-construct residual covariances with elevated modification indices were incorporated in the final measurement model and retained in both structural models: TCA3–TCA9, TCA2–TCA12, TCA11–TCA12, TCA4–TCA9, TCA4–TCA5, TCA5–TCA9, TCA4–TCA10, TCA9–TCA10, TCA7–TCA8, AS_MI9–AS_MI10, AS_MI7–AS_MI19, AS_MI6–AS_MI12, AS_MI4–AS_MI6, AS_MI4–AS_EV4, AS_MI7–AS_EV7, AS_EV8–AS_EV9, AS_MI3–AS_EV3, TCA5–TCA10, AS_MI9–AS_EV9, AS_MI6–AS_EV13, AS_MI12–AS_EV12, and AS_MI6–AS_EV16. These residual covariances were data-informed rather than prespecified a priori, and the same residual-covariance structure was used in the measurement model and both structural models. The complete residual-covariance specification is reported in Supplementary Table S2. The diagonally weighted least squares estimator (DWLS), recommended for ordinal variables, was employed. Model fit was evaluated using χ^2^, the comparative fit index (CFI), Tucker–Lewis index (TLI), root mean square error of approximation (RMSEA), and standardised root mean square residual (SRMR). These indices were considered jointly as complementary indicators of model fit rather than applying any single cut-off value mechanically (Hu & Bentler, 1999; Marsh et al., 2004). Each structural model contained 67 freely estimated model parameters, excluding item thresholds, and 793 degrees of freedom. Item thresholds were additional freely estimated parameters under the ordinal specification and were not included in the 67-parameter count. Models A and B were treated as alternative, non-nested structural specifications. Their fit was compared descriptively; no nested-model chi-square difference test was applied, and no claim of statistical or empirical equivalence was made. Similarity in descriptive fit indices was not interpreted as evidence favouring a particular causal direction. The items included in the final measurement model, together with their codes and the original instruments, are presented in Table S1 of the Supplementary Materials.

The administered EAT-derived and FMPS forms already contained seven response options and were therefore not rescaled for the analyses. Only LSAS item responses were linearly relabelled before CFA/SEM according to \(y = 1 + 2x\), mapping the original response codes 0, 1, 2, and 3 onto 1, 3, 5, and 7, respectively. This relabelling preserved the four ordered LSAS response categories and did not create seven response categories. Its purpose was to harmonise the numerical endpoints across the administered questionnaires and was not required for ordinal estimation. No total or subscale scores were transformed or used as indicators in the CFA or SEM.

## 3. Results

Descriptive statistics were calculated as mean item scores using the indicators retained for each construct. In the final analytical sample (N = 705), the mean score was 2.63 (SD = 1.18) for disordered eating behaviours (ED), 2.38 (SD = 0.95) for social anxiety symptoms (SA), and 2.82 (SD = 1.49) for concern over mistakes (COM).

Firstly, a confirmatory factor analysis (CFA) was estimated to evaluate the overall fit of the measurement model, specifying three latent constructs: ED, SA, and COM. Item 14 from the avoidance (EV) subscale (AS_EV14) was removed because its association with the latent SA factor was 0.246, below the 0.30 criterion applied for retention. The final measurement model therefore comprised 40 reflective indicators: 12 ED indicators (TCA1–TCA12), four COM indicators (P9_PRE, P12_PRE, P13_PRE, and P14_PRE), 13 SA fear indicators, and 11 SA avoidance indicators. The identity and standardised loading of each retained indicator are reported in Supplementary Table S1. The final measurement model incorporated the 22 residual covariances described in the Statistical Analysis subsection and reported in Supplementary Table S2. The model showed the following fit to the data: χ^2^(715) = 1856.671, CFI = 0.954, TLI = 0.950, RMSEA = 0.043, and SRMR = 0.065. Omega (ω) estimates for the three constructs are presented in Table 1.

AVE was 0.486 for ED, 0.402 for SA, and 0.633 for COM; ED and SA were below the commonly used 0.50 criterion. Omega was 0.853, 0.887, and 0.845, respectively, indicating satisfactory reliability. Thus, evidence of convergent validity was weaker for ED and SA than for COM and should be interpreted cautiously.

Positive correlations were observed among the three latent variables. Specifically, SA was associated with ED (r = 0.344, 95% CI [0.270, 0.419]) and with COM (r = 0.390, 95% CI [0.319, 0.461]). Likewise, COM was associated with ED (r = 0.391, 95% CI [0.318, 0.464]).

Discriminant validity between the constructs in the measurement model was assessed using the criterion proposed by Rönkkö and Cho (2022). Comparisons between the measurement model and models constraining the corresponding latent correlation to 0.90 indicated significantly better fit for the unconstrained measurement model: χ^2^diff(1) = 228.11, *p* < 0.001 for ED and SA; χ^2^diff(1) = 213.39, *p* < 0.001 for ED and COM; and χ^2^diff(1) = 233.08, *p* < 0.001 for COM and SA.

### 3.1. Structural Models: Alternative Specifications

In both structural specifications, concern over mistakes was included in the indirect structural association between the two domains, and sociodemographic variables (age and sex) were included as statistical controls.

However, the two models differed in the direction of the primary association between social anxiety symptoms and disordered eating behaviours.

Model A: This model specified an association from disordered eating behaviours to social anxiety symptoms, including concern over mistakes in the indirect association. This model evaluates whether disordered eating behaviours may be associated with higher levels of social anxiety symptoms.

Model B: This model specified an association from social anxiety symptoms to disordered eating behaviours, likewise, including concern over mistakes in the indirect association. This model allows exploration of whether social anxiety symptoms may be linked to higher levels of disordered eating behaviours.

The comparison of these two specifications was conducted to evaluate the robustness and consistency of the observed patterns of association and to determine whether the standardised indirect association through concern over mistakes remained significant regardless of the assumed direction of the primary relationship. This strategy allows the indirect associations to be examined across alternative structural specifications without implying temporal or causal mediation.

#### 3.1.1. Models A and B Showed Very Similar Descriptive Fit Indices

##### Model A: Structural Model with Indirect Paths

**Figure 1:**
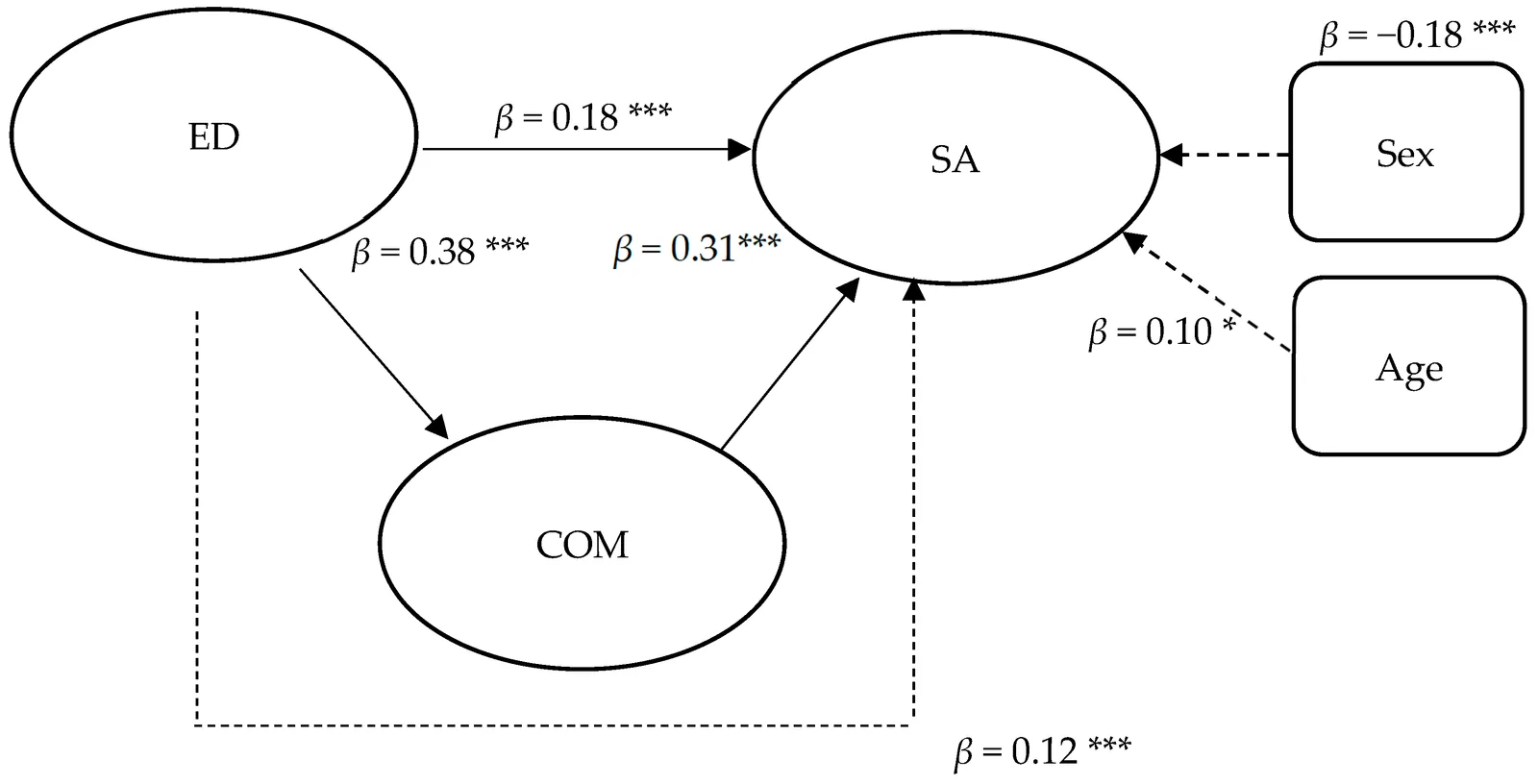
Model A: Indirect association between concern over mistakes, ED, and SA. *Note*. Arrows represent estimated structural associations and do not imply temporal precedence. CV = Control Variable. *p* < 0.05 (*); *p* < 0.001 (***). Solid lines denote direct structural associations; dashed arrows from Age and Sex denote covariate associations; the dashed line below COM denotes the indirect association.

Model A examined the relationship between disordered eating behaviours and social anxiety symptoms, with concern over mistakes included in the indirect structural association. Model A showed the following fit: χ^2^(793) = 2157.046, CFI = 0.947, TLI = 0.947, RMSEA = 0.044, and SRMR = 0.064. At the structural level, ED was positively associated with COM (β = 0.382; 95% CI [0.309, 0.455]), and COM was positively associated with SA (β = 0.306; 95% CI [0.226, 0.386]) (see Figure 1). ED also showed a direct positive association with SA (β = 0.184; 95% CI [0.102, 0.266]). The standardised indirect association of ED with SA through COM was β = 0.117 (95% CI [0.079, 0.154]). Age (β = 0.099, *p* = 0.014) and sex (β = −0.180, *p* < 0.001) were significantly associated with SA. Model parameters are presented in Table 2.

##### Model B: Alternative Structural Model with Indirect Paths

**Figure 2:**
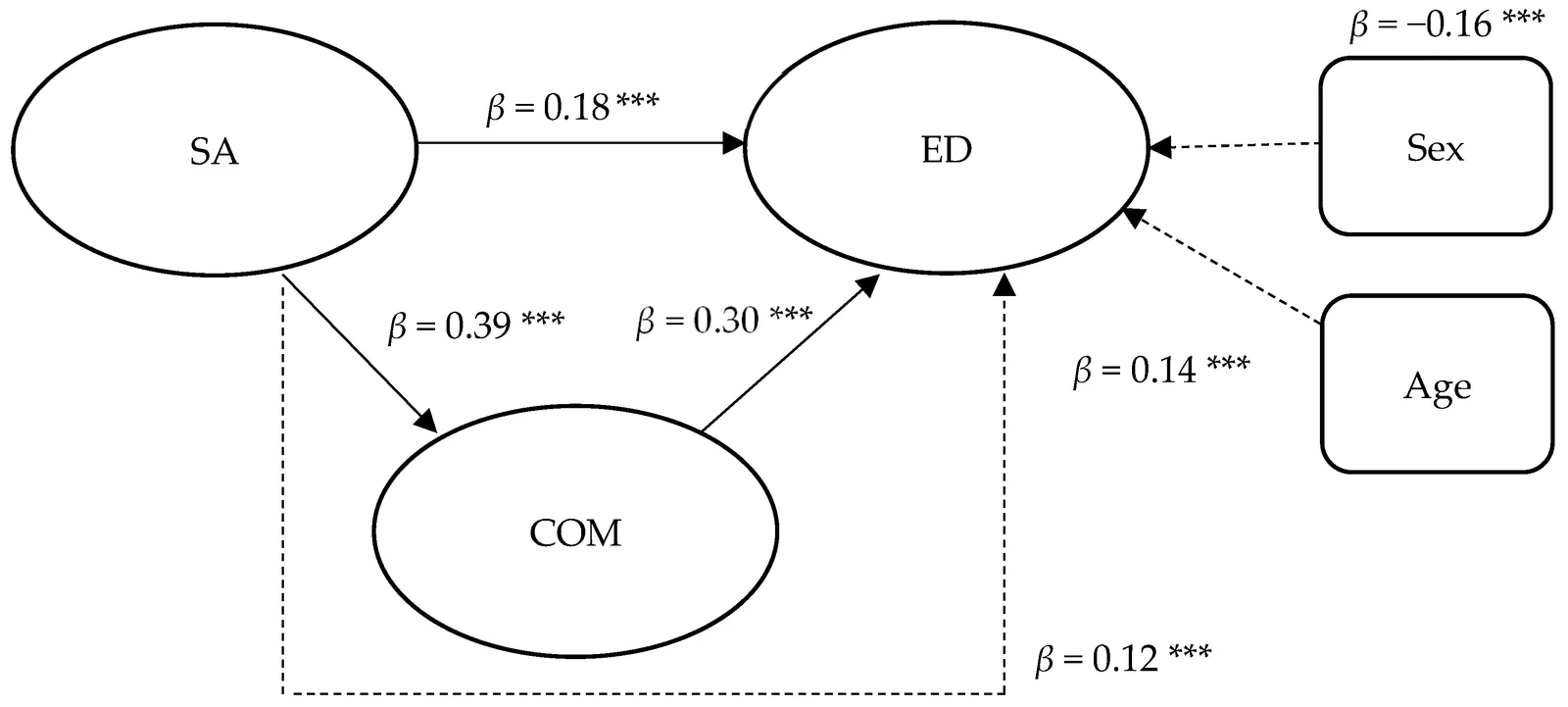
Model B: Indirect association between concern over mistakes, ED, and SA. *Note*. Arrows represent estimated structural associations and do not imply temporal precedence. CV = Control Variable. *p* < 0.001 (***). Solid lines denote direct structural associations; dashed arrows from Age and Sex denote covariate associations; the dashed line below COM denotes the indirect association.

Model B examined the relationship between social anxiety symptoms and disordered eating behaviours, with concern over mistakes included in the indirect structural association. Model B showed the following fit: χ^2^(793) = 2209.502, CFI = 0.946, TLI = 0.947, RMSEA = 0.044, and SRMR = 0.064. SA was positively associated with COM (β = 0.385; 95% CI [0.313, 0.457]), and COM was positively associated with ED (β = 0.302; 95% CI [0.222, 0.381]) (see Figure 2). SA also showed a direct positive association with ED (β = 0.185; 95% CI [0.103, 0.266]). The standardised indirect association of SA with ED through COM was β = 0.116 (95% CI [0.079, 0.154]). Age (β = 0.137, *p* < 0.001) and sex (β = −0.160, *p* < 0.001) were significantly associated with ED. Model parameters are presented in Table 3.

### 3.2. Indirect Associations

The analyses indicated that the indirect associations through concern over mistakes were statistically significant in both structural specifications. Overall, these results show consistent indirect statistical associations involving concern over mistakes across the two alternative structural specifications (see Table 2 for Model A and Table 3 for Model B). The similarity of these indirect associations across both models does not establish temporal precedence, causal mediation, or a preferred direction of association between social anxiety symptoms and disordered eating behaviours.

## 4. Discussion

The present study examined the association between social anxiety symptoms and disordered eating behaviours, as well as the role of concern over mistakes, a central subdimension of maladaptive perfectionism, in this relationship. Using structural equation modelling with alternative specifications, the results indicate that both domains are closely related and that concern over mistakes emerges as a consistently associated cognitive dimension within the observed pattern of relationships.

Firstly, the findings are consistent with the literature documenting a high co-occurrence between social anxiety and eating disorders (Alcaraz-Ibáñez et al., 2023; Kerr-Gaffney et al., 2018). The similar descriptive fit observed across the two structural specifications indicates that the present cross-sectional data do not favour a particular directional specification. Independently, previous longitudinal research has reported prospective associations in both directions between anxiety symptoms and eating-related symptoms over time (Puccio et al., 2017; Trompeter et al., 2025), while other studies have proposed social phobia as a vulnerability factor for eating disorders (Cardi et al., 2018; Ho et al., 2024). These findings from previous research provide relevant context but should not be interpreted as evidence that the present cross-sectional models demonstrate bidirectionality.

Secondly, concern over mistakes showed direct associations with both variables, reinforcing the transdiagnostic perspective of maladaptive perfectionism (Behar et al., 2014; Bernabéu-Brotóns & Marchena-Giráldez, 2022). This pattern is consistent with the multidimensional conceptualisation of perfectionism proposed by Frost et al. (1990), in which concern over mistakes represents a core dimension linked to self-criticism and sensitivity to failure. Furthermore, the results converge with previous evidence linking perfectionistic concerns both to eating disorder symptomatology (Stackpole et al., 2023; Vervaet et al., 2021) and to social anxiety, particularly in dimensions related to negative evaluation (Ferber et al., 2024).

Importantly, the indirect associations estimated through concern over mistakes were significant under both structural specifications, showing a consistent statistical association involving this cognitive dimension and the overlap between social anxiety symptoms and disordered eating behaviours. This finding is consistent with recent network analyses showing that, although eating disorder symptoms may constitute the most central nodes in the network, perfectionistic traits function as bridges connecting different symptom domains (Claus et al., 2025). Given the cross-sectional nature of the data, these indirect associations should be interpreted as statistical associations compatible with a shared psychological vulnerability, rather than as evidence of temporal or causal mediation (Rijnhart et al., 2021; Schuler et al., 2025). This interpretation aligns with previous work highlighting the role of evaluative fears and related constructs (e.g., social appearance anxiety or fear of negative evaluation) as vulnerabilities linking social anxiety and eating-related symptoms (Heimberg et al., 2014; Jain & Sudhir, 2010; Levinson et al., 2013). Similarly, other studies have identified perfectionism, and particularly perfectionistic concerns, as a cross-cutting factor involved in multiple forms of internalising psychopathology, both in longitudinal studies and in more recent meta-analyses (Brosof & Levinson, 2017; Kehayes et al., 2019; Smith et al., 2022).

The present findings identify concern over mistakes as a correlate of both symptom domains. Previous intervention literature supports further research on transdiagnostic interventions targeting shared cognitive vulnerabilities (Egan et al., 2011; Robinson & Wade, 2021). Consistent with the literature linking maladaptive perfectionism to multiple forms of psychopathology, strategies aimed at increasing tolerance of mistakes and reducing self-criticism have been proposed as potentially relevant to simultaneously reducing vulnerability to both anxiety and eating-related symptoms (Barlow & Long, 2023; Lloyd et al., 2019). In line with this perspective, systematic reviews and intervention studies suggest that addressing maladaptive perfectionism may have beneficial effects across multiple symptomatic domains, including social anxiety (Abdollahi et al., 2021; Galloway et al., 2022; Suh et al., 2019). However, the present cross-sectional analyses do not establish that modifying concern over mistakes would produce changes in either social anxiety symptoms or disordered eating behaviours. Such intervention effects would need to be evaluated in longitudinal or experimental studies.

This study should be interpreted in light of several limitations. First, the cross-sectional design prevents the establishment of temporal precedence among the constructs examined. Second, the use of self-report measures may introduce response biases. Third, the use of a non-clinical sample limits the generalisability of the findings to populations with formal diagnoses. Fourth, because item selection and measurement-model evaluation were not independently cross-validated, the resulting measurement specification may be sample-dependent and should be replicated in an independent sample. Fifth, because data were collected collectively in classrooms and the analytical dataset did not retain verified identifiers allowing the clustering structure to be reconstructed, possible within-classroom or within-institution dependence could not be evaluated or statistically adjusted for. The reported estimates should therefore be interpreted with this limitation in mind. In addition, the use of convenience sampling and the lack of systematically recorded participation and refusal rates may have introduced selection bias and limited the representativeness of the sample. The restriction of the sample to students aged 18–25 years may further limit the generalisability of the findings. The use of study-specific reduced item sets and the numerical relabelling of LSAS response categories may also limit direct comparability with the original scoring formats of the parent instruments. Finally, the statistically significant indirect associations observed in the present study should not be interpreted as evidence of temporal or causal mediation. Future research incorporating longitudinal or experimental designs would help clarify the temporal sequence of these associations and allow a more precise evaluation of the mechanisms involved.

Overall, the findings support a transdiagnostic interpretation in which concern over mistakes is consistently associated with both social anxiety symptoms and disordered eating behaviours and may represent a shared psychological vulnerability. Given the cross-sectional design of the study, this evidence should be interpreted as indicative of a shared psychological link rather than proof of direct causal relationships, and it aligns with the literature on comorbidity and transdiagnostic psychological vulnerabilities.

Future studies should examine whether these patterns are replicated across diverse cultural contexts, as previous research has reported variations in the joint expression of social anxiety, perfectionism, and eating-related risk (Brosof et al., 2019; Fekih-Romdhane et al., 2024).

## 5. Conclusions

The findings suggest that concern over mistakes is a shared cognitive correlate associated with both social anxiety symptoms and disordered eating behaviours. These results highlight its potential relevance for future research on maladaptive perfectionism, particularly concern over mistakes, in interventions targeting both social anxiety symptoms and eating-related difficulties. The consistency of associations across alternative structural models highlights the limitations of cross-sectional designs for drawing directional conclusions.

## Figures and Tables

**Table 1:** Reliability and convergent validity measures.

Construct	Omega (ω)	AVE
ED	0.853	0.486
SA	0.887	0.402
COM	0.845	0.633

*Note.* ED (disordered eating behaviours); SA (social anxiety symptoms); COM (concern over mistakes). AVE (average variance extracted).

**Table 2:** Structural parameters of Model A (ED → SA).

Path	B	SE	β	z	*p*	95% CI (B)
ED → COM	0.413	0.047	0.382	8.81	<0.001	[0.321, 0.505]
COM → SA	0.319	0.046	0.306	6.87	<0.001	[0.228, 0.410]
ED → SA	0.208	0.048	0.184	4.31	<0.001	[0.113, 0.303]
Age → SA	0.057	0.023	0.099	2.46	0.014	[0.011, 0.102]
Sex → SA	−0.417	0.091	−0.180	−4.57	<0.001	[−0.595, −0.238]

*Note.* B = unstandardised coefficient; SE = standard error of B; β = standardised coefficient; 95% CI refers to B. ED = disordered eating behaviours; SA = social anxiety symptoms; COM = concern over mistakes. Sex was coded as 0 = women and 1 = men; therefore, negative coefficients indicate lower levels of the corresponding outcome among men relative to women. The standardised indirect association ED → COM → SA was β = 0.117, 95% CI [0.079, 0.154].

**Table 3:** Structural parameters of Model B (SA → ED).

Path	B	SE	β	z	*p*	95% CI (B)
SA → COM	0.417	0.047	0.385	8.94	<0.001	[0.326, 0.509]
SA → ED	0.209	0.048	0.185	4.32	<0.001	[0.114, 0.303]
COM → ED	0.314	0.046	0.302	6.79	<0.001	[0.224, 0.405]
Age → ED	0.078	0.023	0.137	3.42	<0.001	[0.033, 0.124]
Sex → ED	−0.369	0.093	−0.160	−3.96	<0.001	[−0.551, −0.186]

*Note*. B = unstandardised coefficient; SE = standard error of B; β = standardised coefficient; 95% CI refers to B. ED = disordered eating behaviours; SA = social anxiety symptoms; COM = concern over mistakes. Sex was coded as 0 = women and 1 = men; therefore, negative coefficients indicate lower levels of the corresponding outcome among men relative to women. The standardised indirect association SA → COM → ED was β = 0.116, 95% CI [0.079, 0.154].

## Data Availability

The data supporting the findings of this study are available from the corresponding author upon reasonable request. The data are not publicly available due to privacy and ethical considerations related to the protection of participants’ personal data.

## Supplementary Materials

The following supporting information can be downloaded at: https://www.mdpi.com/article/10.3390/ejihpe16100163/s1, Table S1: Standardised Factor Loadings for the Reference CFA. Table S2: Residual Covariances Included in the Final Measurement Model.
