# Supplementary material for: The Role of Concern over Mistakes in the Association Between Social Anxiety and Disordered Eating Behaviours: A Structural Equation Modelling Study
Source: Eur J Investig Health Psychol Educ. 2026 Oct 14;16(10):163. doi: 10.3390/ejihpe16100163 (PMC13703356; doi:10.3390/ejihpe16100163)
Supplement: Supplementary file 1 [file ejihpe-16-00163-s001.zip › ejihpe-4583113-supplementary.pdf]

Table S1

Standardised Factor Loadings for the Reference CFA

Factor	Indicator	λ std.all	SE	95% CI
ED	TCA1	0.722	0.022	[0.679, 0.765]
ED	TCA2	0.797	0.019	[0.759, 0.834]
ED	TCA3	0.498	0.031	[0.437, 0.558]
ED	TCA4	0.508	0.033	[0.444, 0.572]
ED	TCA5	0.636	0.030	[0.577, 0.696]
ED	TCA6	0.902	0.014	[0.875, 0.928]
ED	TCA7	0.796	0.018	[0.760, 0.831]
ED	TCA8	0.812	0.018	[0.777, 0.847]
ED	TCA9	0.438	0.034	[0.373, 0.504]
ED	TCA10	0.560	0.031	[0.500, 0.621]
ED	TCA11	0.790	0.022	[0.747, 0.834]
ED	TCA12	0.752	0.023	[0.707, 0.797]
SA	AS_MI1	0.727	0.028	[0.671, 0.782]
SA	AS_MI3	0.738	0.035	[0.669, 0.808]
SA	AS_MI4	0.664	0.041	[0.585, 0.743]
SA	AS_MI6	0.514	0.031	[0.452, 0.575]
SA	AS_MI7	0.709	0.031	[0.649, 0.769]
SA	AS_MI9	0.801	0.019	[0.763, 0.839]
SA	AS_MI10	0.758	0.024	[0.710, 0.805]
SA	AS_MI12	0.763	0.021	[0.722, 0.803]
SA	AS_MI15	0.730	0.024	[0.683, 0.778]
SA	AS_MI17	0.651	0.028	[0.596, 0.705]
SA	AS_MI18	0.694	0.030	[0.635, 0.752]
SA	AS_MI19	0.642	0.032	[0.578, 0.706]
SA	AS_MI20	0.691	0.028	[0.637, 0.746]
SA	AS_EV2	0.525	0.036	[0.454, 0.595]

Factor	Indicator	λ std.all	SE	95% CI
SA	AS_EV3	0.507	0.038	[0.433, 0.581]
SA	AS_EV4	0.431	0.049	[0.334, 0.527]
SA	AS_EV5	0.545	0.034	[0.479, 0.611]
SA	AS_EV7	0.447	0.040	[0.369, 0.525]
SA	AS_EV8	0.669	0.027	[0.615, 0.722]
SA	AS_EV9	0.697	0.025	[0.647, 0.747]
SA	AS_EV11	0.411	0.041	[0.330, 0.491]
SA	AS_EV12	0.538	0.033	[0.473, 0.604]
SA	AS_EV13	0.515	0.032	[0.453, 0.577]
SA	AS_EV16	0.599	0.029	[0.542, 0.656]
COM	P9_PRE	0.834	0.022	[0.791, 0.877]
COM	P12_PRE	0.879	0.018	[0.844, 0.914]
COM	P13_PRE	0.821	0.021	[0.780, 0.861]
COM	P14_PRE	0.625	0.030	[0.568, 0.683]

Note. ED = disordered eating behaviours; SA = social anxiety symptoms; COM = concern over mistakes. The SA indicators comprise 13 fear items (AS_MI) and 11 avoidance items (AS_EV). All indicators were modelled as reflective indicators, and all retained standardised loadings were statistically significant ($p < .001$). AS_EV14 was removed from the final measurement model because its association with the latent SA factor was .246, below the .30 criterion applied for retention. Item codes correspond to the variable names used in the analytical dataset and do not necessarily correspond to the original numbering of the parent instruments.

Table S2

Residual Covariances Included in the Final Measurement Model

Residual pair	Estimate	SE	Standardised	95% CI
TCA3–TCA9	-0.089	0.033	-0.114	[-0.154, -0.024]
TCA2–TCA12	-0.102	0.023	-0.257	[-0.148, -0.057]
TCA11–TCA12	0.199	0.026	0.494	[0.148, 0.251]
TCA4–TCA9	0.319	0.029	0.412	[0.263, 0.375]
TCA4–TCA5	0.253	0.028	0.381	[0.198, 0.309]
TCA5–TCA9	0.321	0.030	0.462	[0.261, 0.380]
TCA4–TCA10	0.222	0.028	0.312	[0.168, 0.277]
TCA9–TCA10	0.251	0.029	0.337	[0.195, 0.307]
TCA7–TCA8	0.097	0.020	0.275	[0.058, 0.137]
AS_MI9–AS_MI10	0.195	0.023	0.498	[0.150, 0.240]
AS_MI7–AS_MI19	0.207	0.034	0.383	[0.140, 0.274]
AS_MI6–AS_MI12	0.112	0.026	0.203	[0.062, 0.163]
AS_MI4–AS_MI6	-0.166	0.047	-0.259	[-0.259, -0.073]
AS_MI4–AS_EV4	0.538	0.044	0.797	[0.452, 0.623]
AS_MI7–AS_EV7	0.365	0.035	0.579	[0.298, 0.433]
AS_EV8–AS_EV9	0.225	0.026	0.422	[0.174, 0.276]

Residual pair	Estimate	SE	Standardised	95% CI
AS_MI3–AS_EV3	0.353	0.038	0.607	[0.278, 0.428]
TCA5–TCA10	0.217	0.030	0.339	[0.159, 0.275]
AS_MI9–AS_EV9	0.215	0.025	0.501	[0.166, 0.264]
AS_MI6–AS_EV13	0.190	0.028	0.259	[0.135, 0.246]
AS_MI12–AS_EV12	0.263	0.026	0.483	[0.212, 0.314]
AS_MI6–AS_EV16	0.183	0.026	0.267	[0.132, 0.235]

Note. The 22 residual covariances shown were incorporated in the final measurement model after examination of elevated modification indices and were retained in both structural models. The same residual-covariance structure was used in the measurement model and in both structural models.
